# Optimized intrusion detection using particle swarm optimization and neural networks in simulated and physical network environments

**DOI:** 10.1016/j.mex.2026.103824

**Published:** 2026-02-17

**Authors:** Vaishnavi Ganesh, SV Deshmukh

**Affiliations:** a Priyadarshini College of Engineering, Nagpur, India; b Dr. D. Y. Patil Institute of Technology(DIT), Pimpri, Pune, Maharashtra, India

**Keywords:** Intrusion detection system, Particle swarm optimization, Decision trees, K-nearest neighbor, Artificial Neural Networks, Denial of Service attacks, Man-in-the-middle

## Abstract

This method article presents a reproducible framework for constructing and evaluating an intrusion detection system using optimized machine learning workflows. Network traffic was collected by implementing two independently controlled setups: a software-based network model and a small-scale real hardware network, with packet traces recorded using Wireshark. These traces were merged with widely used reference datasets to ensure coverage of diverse attack behaviors. Feature dimensionality was reduced using Particle Swarm Optimization, enabling efficient learning while limiting redundant network attributes. The refined feature sets were subsequently used to train multiple classification models, including tree-based, distance-based, and neural learning approaches. Experimental evaluation shows that the neural model trained on PSO-selected features delivers the most stable detection behavior, combining strong classification performance with a consistently low false alert rate, thereby validating the proposed method for intrusion analysis studies.

• Generation of packet-level datasets from independently built virtual and physical network environments

• Optimization-driven feature selection using Particle Swarm Optimization to reduce redundancy

• Cross-model evaluation of optimized classifiers, demonstrating superior reliability of the PSO-enhanced neural approach

## Graphical abstract

**Figure:**
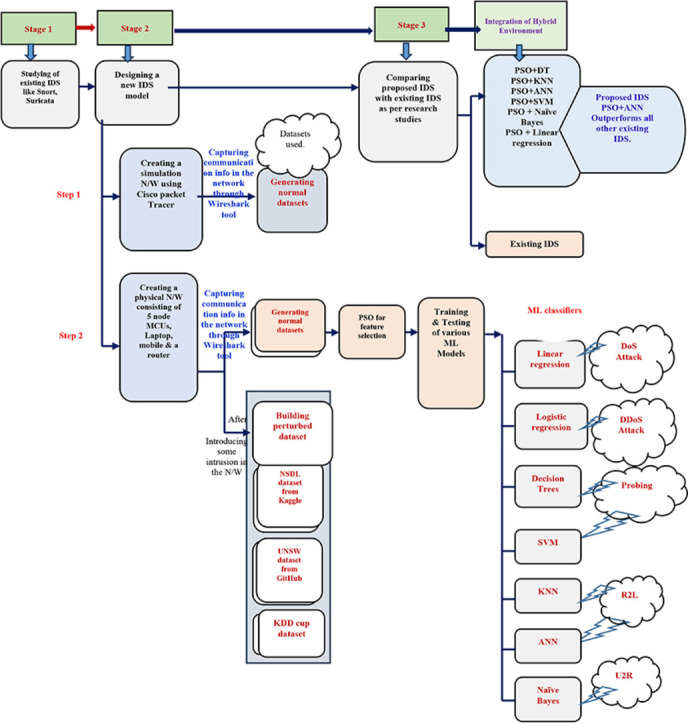

## Specifications table

**Table:** 

**Subject area**	Computer Science
**More specific subject area**	Network Security
**Name of your method**	Implementing Intrusion Detection System
**Name and reference of original method**	Intrusion Detection Systems for Wireless Sensor Networks using Computational Intelligence Techniques
**Resource availability**	None

## Background

The primary motivation behind this research is to enhance the security of organizational databases against external threats and unauthorized intrusions. In numerous real-world incidents, cyber attackers have successfully infiltrated sensitive networks—particularly in sectors such as banking—gaining illegal access to critical databases and compromising confidential information, including customer passwords and financial records. Such breaches often lead to significant financial losses and erosion of public trust, especially among innocent customers who fall victim to these attacks.

Recognizing the growing frequency and sophistication of these intrusions, this research was initiated with the aim of developing an improved Intrusion Detection System (IDS) capable of identifying and mitigating malicious activity with higher accuracy and lower false alarm rates. While various IDS solutions already exist in the market, many still suffer from limitations such as delayed detection, high false positives, or inability to detect novel attack patterns. This work seeks to address those gaps by designing a more efficient and responsive IDS framework, focused on protecting sensitive organizational assets from evolving cybersecurity threats.

## Method details

**Fig. 1:**
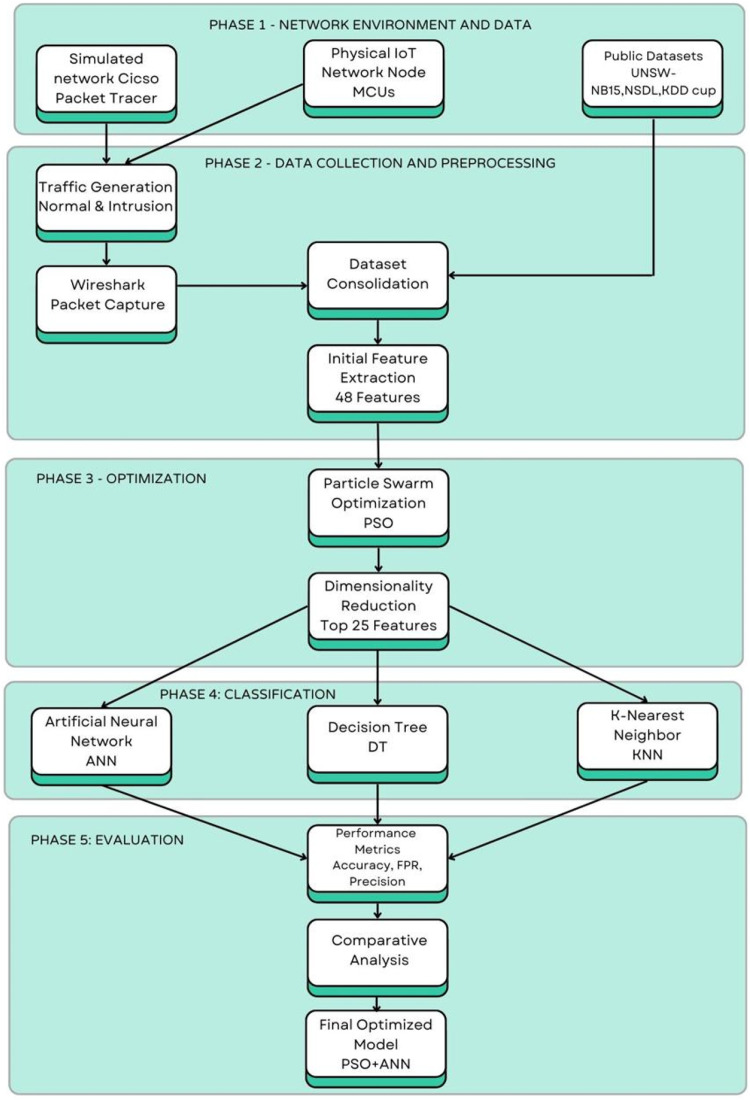
Flowchart of the proposed IDS.

The gist of the entire research work is explained in Fig. 1.

1. Here the first phase consists of creating networks. First a simulation network is created in Cisco Packet Tracer and the communication that takes place among the various source and destination nodes is acquired with the help of Wireshark tool. Also an actual physical network has been created in the research work for obtaining more datasets of packet communication between the various nodes in the network using Wireshark tool. Some kinds of intrusions have been induced in the network and again the intrusion induced dataset was obtained.
2. All these datasets along with some standard datasets like NSDL, UNSW and kdd cup dataset are used for training various machine learning algorithms.
3. Prior to that, Particle swarm optimization algorithm was used for feature selection of the obtained datasets. It approximately reduced 48 total features to 25 features.
4. Now these datasets with reduced number of features are used for training of various machine learning algorithms.
5. The performance parameters were compared for variuos combinations of the proposed algorithm of IDS, like for PSO+ANN, PSO+DT and PSO+kNN. And it was found that PSO+ANN outperformed the other two. Also the proposed IDS combination of PSO+ANN outperformed other commercially existing IDS with highest detection rate and lowest false positive rate.

### Simulated and physical network setup for dataset generation

The research involves the creation of two distinct network environments for the purpose of capturing real-world communication data and generating meaningful datasets for machine learning-based intrusion detection.

• The first is a simulated enterprise network, developed using Cisco Packet Tracer, consisting of interconnected PCs, switches, and routers organized in a LAN architecture. This simulated setup emulates typical enterprise-level network behavior and traffic flow under controlled conditions [6].
• The second is a physical network setup, comprising a 5-node Microcontroller Unit (MCU) cluster, a laptop, and a mobile hotspot. These devices were physically interconnected to replicate a minimal real-world deployment scenario with live wireless communication [7].

**Fig. 2:**
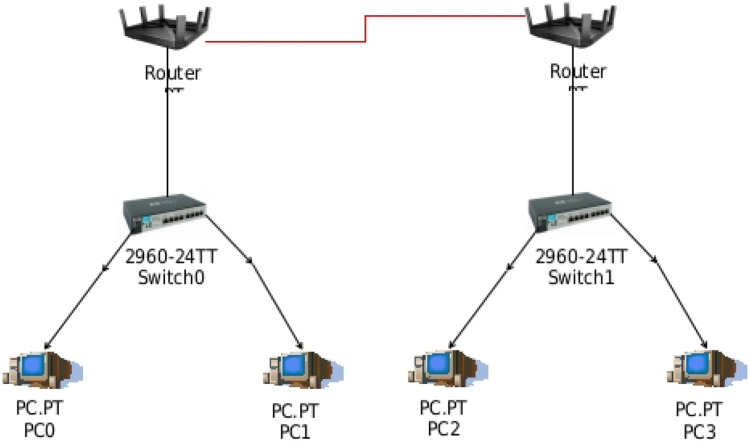
Showing the test network architecture in Cisco packet tracer.

Both environments were subjected to normal operations as well as induced intrusion events. Network activity was continuously monitored and captured using the Wireshark tool, which recorded packet-level data including source and destination IPs, protocols, and traffic patterns. The datasets generated from both the simulated and physical setups were later preprocessed and used to train multiple machine learning algorithms for intrusion detection Fig. 2 Showing the simulation network created in Cisco Packet Tracer.

### Intrusion pattern identification from network simulation and wireshark capture

1. Simulated Network Construction
  A simulated enterprise network environment was created using Cisco Packet Tracer, comprising interconnected PCs, switches, and routers via LAN connections [8]. This setup emulates real-time communication across devices in an organizational network, as shown in Fig. 4 [1].
2. Packet Capture and Data Collection
  Using the Wireshark tool, approximately 10 min of live network activity on a laptop system was recorded. The captured packets included all communication traffic from the simulation environment [9]. Key attributes such as source and destination IP addresses, communication protocols, and packet transmission durations were extracted for analysis [2].
3. Protocol-Wise Delay Analysis and Intrusion Hypothesis
  The captured data was analyzed protocol-wise using filters in Wireshark. Communication durations between source and destination IPs were plotted as timing graphs [10]. The destination exhibiting the longest response time was identified as the most resistant to the incoming traffic. A significantly longer delay from a particular source to this destination may suggest unusual behavior or a potential intrusion attempt, as the network delays indicate possible rejection or filtering by the destination node [1].
4. Identification of Suspicious Source IPs
  To trace the origin of potential anomalies, filters were applied to isolate communication related to the identified high-resistance destination IP [11]. The source IP occupying the maximum duration in the graph was flagged as suspicious. This IP was hypothesized to be the origin of the anomalous or malicious packet, potentially indicating the presence of an intruder or a DoS attempt [12].

**Fig. 3:**
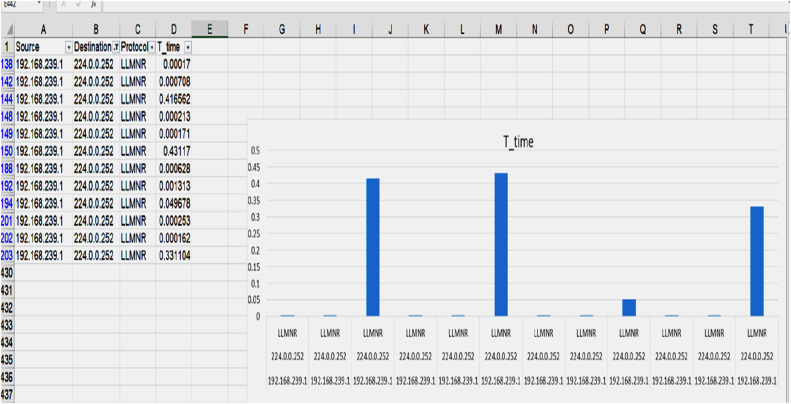
Showing the amount of time, it takes for packets to go between a source IP and a destination IP.

The information is shown in the following R-plotted graph in Fig. 3.

The graph is generated based on multiple analytical criteria, including variations in source IP addresses, destination IP addresses, the communication protocols involved, and the time duration required for a specific protocol to influence a particular destination IP before any observable impact is reflected back on the corresponding source IP [13].

### Dataset development from physical network using node MCUs and iot devices

In addition to the simulated environment, a physical network was constructed using six Node MCUs, a laptop, and a mobile device connected via a mobile hotspot-based internet connection [14]. Each Node MCU was interfaced with an LED component, and the network was configured to simulate typical IoT device communication.

Initially, normal activity was simulated by sending standard ON and OFF commands to the LEDs from a mobile application programmed for IoT control [15]. These interactions were captured using the Wireshark tool, forming the basis of the normal dataset [1].

To simulate malicious behavior, the IP address of one of the Node MCUs was intentionally modified to a malformed or unusually long string, introducing an element of suspicious network behavior [1,2]. Additionally, to simulate potential intrusion patterns, a sequence of two consecutive ON commands, followed by a two-second delay and two OFF commands, was sent to a node [16]. The traffic generated during this activity was again recorded using Wireshark, resulting in an intrusion-induced dataset.

These custom datasets—both normal and intrusion-based—were used to train the neural network model. Supplementary datasets were also sourced from UNSW-NB15, as well as publicly available repositories on Kaggle and GitHub for comparative evaluation and extended training [1].

### Physical network configuration and dataset sources

**Fig. 4:**
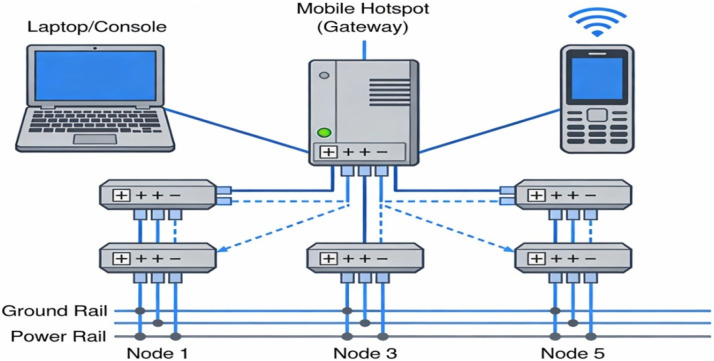
Actual physical network created using end users, console devices and 5 node MCUs connected mobile network.

A physical wireless network was constructed using a laptop, a mobile device, and five Node MCUs, as depicted in Fig. 4. This setup facilitated the generation of network traffic under both legitimate and malicious conditions, which was recorded and used for the development of multiple datasets [1].

The datasets employed in this research include:

i. Simulated Normal Dataset: Captured using the Wireshark tool from a simulated enterprise network created in Cisco Packet Tracer [17]. This dataset reflects typical communication patterns under controlled, attack-free conditions.
ii. IoT-Based Normal Dataset: Generated from the physical Node MCU setup, where standard ON/OFF commands were transmitted to simulate normal IoT communication behavior [18].
iii. NSL-KDD Dataset: Sourced from Kaggle, this benchmark dataset contains a wide range of labeled intrusion types and is widely used for training machine learning models in IDS research [19].
iv. Intrusion-Induced IoT Dataset: A perturbed dataset was developed by introducing irregular behaviors and traffic anomalies within the IoT network, such as malformed IP addresses and abnormal command sequences [1].
v. UNSW-NB15 Dataset: Downloaded from GitHub, this dataset includes complex network traffic data generated by various malware and attack types, representing contemporary intrusion scenarios [20].
vi. KDD CUP 99 Dataset: A classical benchmark dataset consisting of connection-level records representing normal and attack behaviors in a computer network [21].

**Fig. 5:**
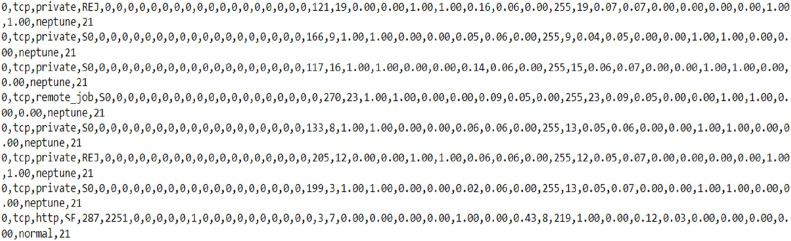
NSDL Datasets used for training various ML models.

**Fig. 6:**
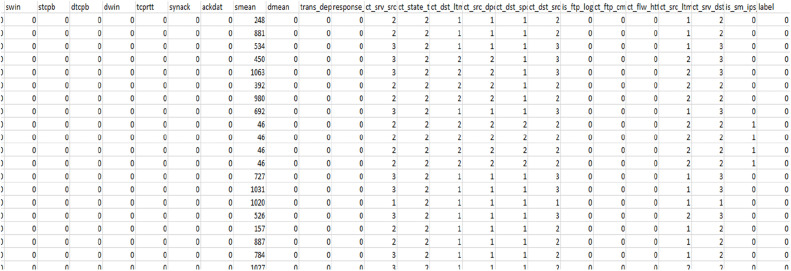
UNSW Datasets used for training various ML models.

These datasets shown in Fig. 5, Fig. 6, form the foundation for training and evaluating the proposed intrusion detection system across both traditional and IoT-based network contexts.

### Feature selection and model training

To enhance model efficiency and reduce computational overhead, Particle Swarm Optimization (PSO) was employed as the feature selection technique across all datasets. Each dataset initially contained 48 features, from which PSO identified the top 25 most relevant features based on their contribution to accurate classification. This reduced feature set was then used to train various machine learning models aimed at detecting network-based intrusions [22].

Multiple machine learning algorithms were trained and evaluated, including Linear Regression, Logistic Regression, Support Vector Machines (SVM), Naïve Bayes, K-Nearest Neighbor (KNN), Decision Trees (DT), and Artificial Neural Networks (ANN) using the datasets shown in Fig. 5, Fig. 6. These models were tested against traffic records containing both normal and malicious behaviors [23]. The types of attacks considered in the classification process included Neptune, Smurf, Pod, Worm, and other known intrusion patterns.

Additionally, Fig. 6 presents a collection of international dataset values that were utilized for training and testing the machine learning models. These datasets provided a diverse range of attack scenarios and traffic characteristics, thereby improving the robustness and generalization capability of the proposed IDS framework [24].

**Fig. 7:**
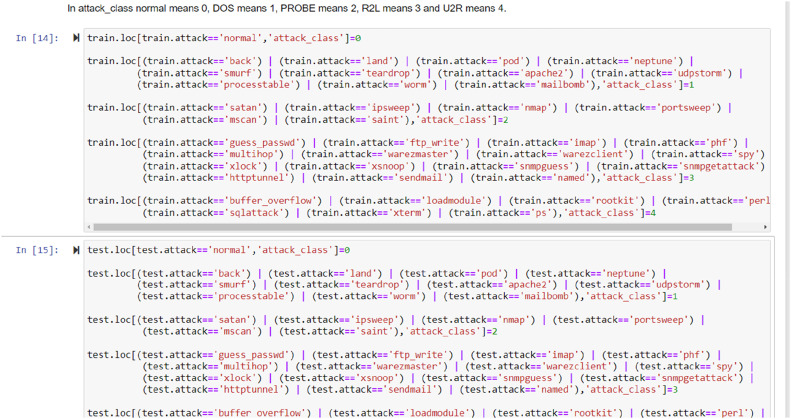
Training given to various Machine Learning Models.

The accompanying illustration demonstrates that a regular situation will be labelled as a 0, whereas an attack of the types such as Neptune, Smurf, Pod, etc., which can be categorized as Denial of Service (DOS) attack will be labelled as 1, Probe attack will be labelled as 2, R2L as 3 andU2R as 4. The Fig. 7 is representing the correlations among the data values which is used in training mode [25].

### Method validation

A denial of service (DOS) assault is fully classified as 1; a probing attack is fully classified as 2, an R2L attack is fully classified as 3, a U2R attack is fully classified as 4, and a normal situation is fully classified as 0. As a result, as seen below, this training is delivered to ML models that can detect attacks with various degrees of accuracy.

**Fig. 8:**
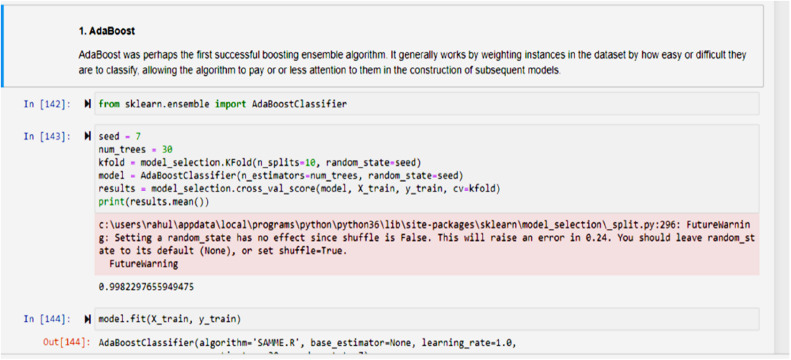
Integration of Adaboost ML sequences with their obatined results based on the seeds.

**Fig. 9:**
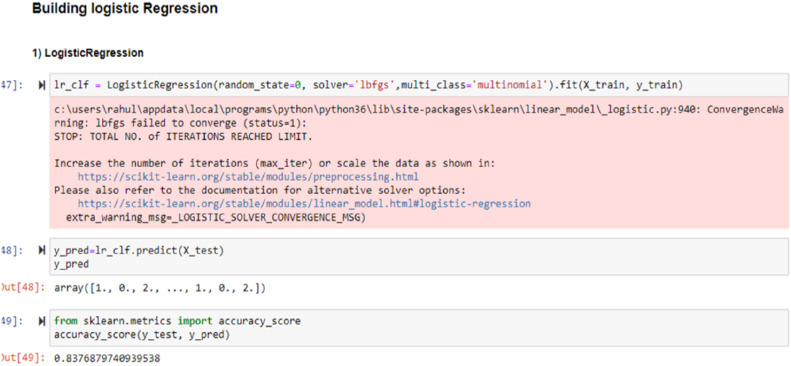
Integration of Logistic Regression sequences with their obtained results based on the communications data seeds.

The Fig. 8 showing the integration of Adaboost-ML sequences with their obtained results based on the seeds. The Fig. 9 showing the integration of Logistic Regression sequences with their obtained results based on the communication data seeds.

Training was given to a variety of Machine Learning models like Adaboost, logistic regression, etc. using the datasets from the research study as shown in Fig. 3 as well as some of the standard datasets like UNSW, kdd cup, and NSDL as shown in Fig. 5, Fig. 6, and their accuracy percentage of detecting attacks is shown below in Fig. 8, Fig. 9.

It was observed that the accuracy of detection rate of Adaboost is 99.82 %.

The proposed IDS research results are compared amongst PSO+DT, PSO+KNN and PSO+ANN, in which it was observed that PSO+ANN had highest precision rate of 99.77 % and lowest false positive rate of 0.02 % as shown in Table 1, Table 2.

**Table 1:** Measurements of performance for proposed classifier.

Measure	PSO+DT ( %)	PSO+KNN ( %)	PSO+ANN ( %)
Predictability	98.7	99.6	99.7
Accuracy	75.2	88.4	90.2
Low Statistical Value (NPV)	99.5	99.8	99.8
F1 Rank	81.7	92.1	94.1

**Table 2:** Assessment of suggested classifications.

Measures	PSO+DT ( %)	PSO+KNN ( %)	PSO+ANN ( %)
Precision	98.5	99.5	99.77
Detection Rate (DR)	89.5	96.1	97.2
False Positive Rate (FPR)	1.2	0.3	0.02

Here is a comparison of PSO with each of the three classifiers: K Nearest Neighbor, ANN, and decision trees (DT) (KNN).

**Fig. 10:**
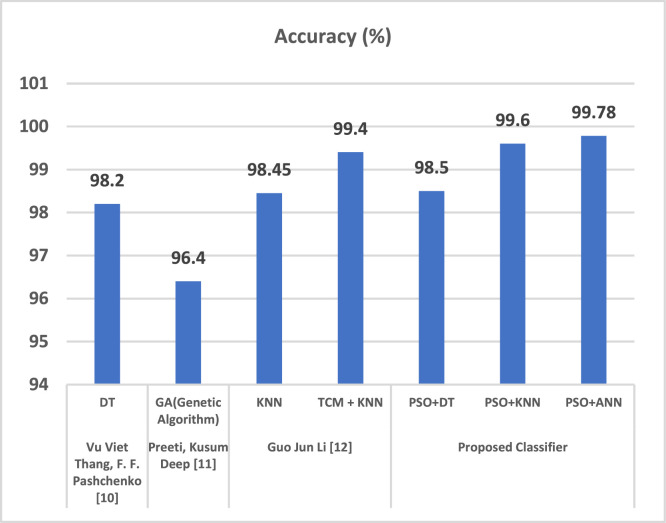
Comparison evaluation of the current system to generate the accuracy assessment.

**Fig. 11:**
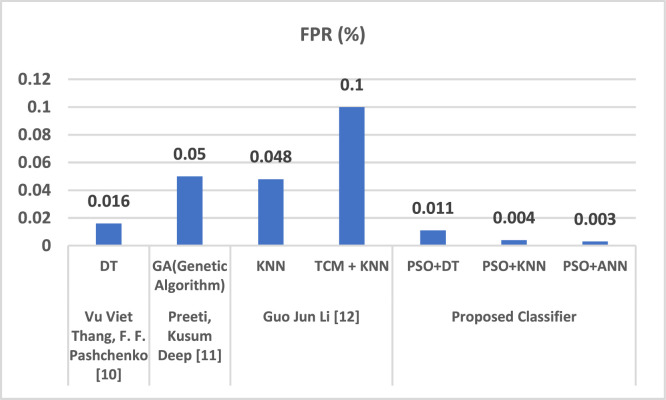
Comparison evaluation of the current system to generate the false positive rate assessment.

Several studies compared the proposed IDS system to the existing IDS projects as shown in Table 3, Fig. 10, Fig. 11.

**Table 3:** Comparison evaluation of the current system.

Authors	Algorithm	Accuracy ( %)	FPR ( %)
Vu Viet Thang, F. F. Pashchenko [3]	DT	98.2	0.016
Preeti, Kusum Deep [4]	GA(Genetic Algorithm)	96.4	0.05
Guo Jun Li [5]	KNN	98.45	0.048
	TCM + KNN	99.4	0.1
Proposed Classifier	PSO+DT	98.5	0.011
	PSO+KNN	99.6	0.004
	PSO+ANN	99.78	0.003

As a result, it can be said that the suggested IDS, PSO+ANN, offers the highest accuracy and the lowest FPR when compared to other systems. In comparison to the other IDS, the Proposed IDS exhibits the best attack detection accuracy i.e. 99.78 %, and the lowest False Positive rate of 0.003 %, as seen in Table 3 and hence it outperformed all the other existing IDS.

This research involved the creation of two distinct network environments: a physical network, and a simulated network constructed using Cisco Packet Tracer. Network traffic was monitored using the Wireshark tool, which captured packets exchanged between various source and destination IP addresses. These captures were used to generate custom datasets. In addition to these, publicly available benchmark datasets such as UNSW-NB15, KDD Cup, and NSL-KDD were also incorporated into the study.

Prior to training, all datasets underwent feature selection using Particle Swarm Optimization (PSO) to reduce dimensionality and improve learning efficiency. Several machine learning models were then trained on the optimized datasets to classify network traffic and detect potential intrusions. These included PSO+Decision Trees (DT), PSO+*K*-Nearest Neighbors (KNN), and PSO+Artificial Neural Networks (ANN). The performance of the proposed IDS was benchmarked against existing IDS models, and it was observed to outperform them in terms of both efficiency and accuracy.

The proposed PSO+ANN model achieved a true positive rate of 99.78 % and a false positive rate as low as 0.003 %, demonstrating strong detection capability. However, the system also has limitations. It is primarily effective within the monitored network and has limited capability in identifying attacks originating from external or upstream networks. Additionally, the use of PSO for feature selection, while improving efficiency, may have led to slight compromises in detection accuracy by excluding certain less frequent but relevant features.

These limitations highlight the need for future research to expand the model’s adaptability to dynamic network structures and to explore alternative optimization techniques that can balance efficiency with maximal detection performance.

### Limitations

While the proposed PSO+ANN-based IDS demonstrates exceptional performance, several limitations should be acknowledged to provide a balanced perspective:

• Scope of Detection Limited to Active Network: The current approach detects intrusions within the actively monitored network but may fail to identify attacks originating from ascendant or upstream networks. This limitation can impact the broader coverage of intrusion detection across multi-layered architectures.
• Impact of Feature Reduction on Classifier Accuracy: While Particle Swarm Optimization (PSO) improves training efficiency by reducing the feature space, it may also lead to the exclusion of subtle but important features, thereby slightly lowering the accuracy of some machine learning classifiers in detecting certain types of attacks.
• Class Imbalance in Rare Attacks: Rare attacks such as R2L and U2R remain harder to detect due to their limited representation in the datasets, which can lead to lower recall despite high overall accuracy.
• Fixed Network Topology in Simulation: The simulated and physical environments use relatively static network configurations. In real-world enterprise and IoT networks, dynamic topologies may introduce new behavior patterns that challenge the model's assumptions.
• No Real-Time Deployment Yet: The current evaluation is conducted in an offline simulation and test environment. Real-time integration, latency handling, and response strategies were not included in this phase of experimentation.

## Ethics statements

All datasets used in this study were either collected in a controlled network environment (simulated or physical) without involving any personal or user-identifiable information, or sourced from publicly available benchmark repositories such as UNSW-NB15, NSL-KDD, and KDD CUP 99, which are anonymized and widely accepted for research purposes.

The custom datasets generated using Wireshark were collected exclusively from a self-created network topology involving Node MCUs, a laptop, and a mobile hotspot, with no real user data or personal information captured at any stage. The data was used solely for academic and experimental purposes, adhering to ethical standards for data privacy and cybersecurity research.

No human participants, sensitive personal data, or external networks were involved in any part of the data collection or experimentation. Therefore, no ethical approval was required under institutional or national guidelines.

## CRediT authorship contribution statement

**Vaishnavi Ganesh:** Conceptualization, Methodology, Validation, Formal analysis, Resources, Writing – original draft, Writing – review & editing, Supervision. **SV Deshmukh:** Conceptualization, Software, Validation, Investigation, Data curation, Writing – original draft, Visualization.

## Declaration of competing interests

The authors declare that they have no known competing financial interests or personal relationships that could have appeared to influence the work reported in this paper.

## Data Availability

Data will be made available on request.
